# A Single-Center, Observational Study Assessing Functional Outcomes After Arthroscopic Anterior Cruciate Ligament Reconstruction Using Suspensory Tibial Fixation With a Polyether Ether Ketone (PEEK) Button

**DOI:** 10.7759/cureus.64779

**Published:** 2024-07-17

**Authors:** Neeraj Adkar, Satwik Thareja, Ravi A Kerhalkar, Prajwal Sadalagi

**Affiliations:** 1 Department of Orthopedics, Saishree Hospital, Pune, IND

**Keywords:** suspensory tibial fixation, polyether ether ketone (peek) button, functional outcomes, femoral fixation, anterior cruciate ligament reconstruction

## Abstract

Background

Anterior cruciate ligament reconstruction (ACLR) is a crucial procedure in orthopedic surgery. This study evaluates the efficacy and safety of ACLR employing suspensory tibial fixation with a polyether ether ketone (PEEK) button.

Methodology

This retrospective observational study conducted at Sai Shree Hospital, Pune, India, between November 2023 and December 2023 enrolled 47 subjects aged 18-60 years who underwent arthroscopic ACLR utilizing the T-Button-A Adjustable Loop Ultra-High-Molecular-Weight Polyethylene Suture PEEK button. The functional outcomes and patient-reported outcomes were assessed using the International Knee Documentation Committee (IKDC) score, the Modified Cincinnati Rating System Questionnaire (MCRS), the Single Assessment Numeric Evaluation (SANE) score, the Tegner Activity Level (TAL) Scale, and Knee Injury and Osteoarthritis Outcome Score (KOOS) quality of life subscale.

Results

Femoral fixation utilized 27 (57.4%) Proloop Ultra Adjustable Loop Button 60 mm, 19 (40.4%) Infiloop Fixed Loop Button 20 mm, and 1 (2.1%) Infiloop Fixed Loop Button 30 mm. Tibial fixation solely relied on T-Button A (PEEK Tibial Button With Adjustable Loop 90 mm). Postoperative evaluations revealed favorable IKDC (79.49 ± 12.67), MCRS (81.32 ± 11.57), SANE (77.83 ± 11.11), TAL Scale (3.87 ± 0.99) and KOOS quality of life subscale (83.81 ± 13.07) scores.

Conclusions

The findings affirm the efficacy and safety of arthroscopic ACLR utilizing suspensory tibial fixation with the PEEK button, supporting its use for improved patient outcomes.

## Introduction

The integrity of the anterior cruciate ligament (ACL) is essential for maintaining knee stability and facilitating the intricate movements required for various physical activities. As a vital ligament connecting the femur to the tibia within the knee joint, the ACL safeguards against anterior tibial translation and rotational instability [1]. However, the incidence of ACL injuries remains substantial, particularly in sports characterized by rapid directional changes and high-impact maneuvers such as soccer, basketball, football, and skiing [2,3].

ACL injuries entail significant morbidity, manifesting as pain, swelling, and functional impairment, adversely affecting individuals’ mobility and athletic performance [4]. Despite advancements in treatment modalities, including physiotherapy and surgical intervention, the long-term sequelae of ACL tears, such as knee osteoarthritis, continue to pose challenges, often necessitating invasive interventions such as total knee replacement [5,6].

Epidemiologically, ACL injuries are a prevalent concern, particularly among athletes, with a significant proportion resulting from non-contact mechanisms [7,8]. These injuries underscore the imperative for preventive strategies, timely diagnosis, and efficacious management to mitigate both immediate and long-term ramifications [9]. Preventive programs emphasizing neuromuscular training and muscle strengthening have shown promise in reducing ACL injury incidence [10].

In India, the prevalence of ACL injuries is noteworthy, especially among athletes and physically active individuals. A retrospective study reported a high incidence rate of ACL injuries among recreational athletes and football players, underscoring the need for tailored preventive measures and effective treatment strategies [7]. Moreover, epidemiological data indicated an annual incidence of ACL tears of 68.6 per 100,000 person-years, with a significant portion requiring surgical reconstruction [9]. These statistics highlight the substantial burden of ACL injuries within the population and emphasize the importance of addressing this issue through comprehensive preventive and therapeutic approaches.

Surgical reconstruction, particularly arthroscopic anterior cruciate ligament reconstruction (ACLR), is pivotal for restoring knee stability and function in ACL tear patients, with advancements aiming to enhance outcomes [11,12]. Notably, suspensory fixation methods, such as polyether ether ketone (PEEK) buttons, are recognized for biomechanical compatibility and bone-to-graft healing facilitation [12]. The properties of PEEK, resembling human bone, offer advantages such as strength and radiolucency in fixation devices [13,14]. Recent studies show positive outcomes with PEEK button fixation, indicating high graft survival and functional recovery rates [15]. However, ongoing research is crucial to refine ACLR techniques for optimal outcomes and patient satisfaction [16,17].

The rationale for conducting this observational study stems from the need to comprehensively evaluate the efficacy and safety of innovative ACLR techniques, particularly those utilizing suspensory tibial fixation with a PEEK button. While previous research has highlighted the potential advantages of this approach, comprehensive data on functional outcomes, patient-reported outcomes, and safety profiles are lacking [18,19]. This study aims to bridge this gap by collecting and analyzing data on the effectiveness of the PEEK button technique, thereby providing valuable insights for clinicians, patients, and healthcare policymakers on its viability as a standard care practice for ACLR.

## Materials and methods

Study design and participants

This retrospective, observational study aimed to comprehensively evaluate the functional outcomes, patient-reported metrics, and safety parameters associated with ACLR utilizing four devices: Infiloop Fixed Loop UHMWPE Suture Titanium Button 20 mm, Infiloop Fixed Loop UHMWPE Suture Titanium Button 30 mm, Proloop Adjustable Loop UHMWPE Suture Titanium Button 60 mm, and T-Button A Adjustable Loop UHMWPE Suture PEEK Button 90 mm. A total of 47 participants, aged 18 to 60 years, were enrolled based on the inclusion criteria, which included those who had undergone an arthroscopic ACLR procedure using the adjustable PEEK button at least one month before their follow-up and were willing to provide informed consent. Exclusion criteria encompassed participants experiencing traumatic knee injury and those who were not willing to attend the follow-up visit. The sample size was determined based on the availability of required data and participants’ consent to participate in the study.

Functional outcomes, assessed using the International Knee Documentation Committee score (IKDC) and Modified Cincinnati Rating System Questionnaire (MCRS), served as primary endpoints. Secondary endpoints included patient-reported outcomes and activity levels measured by the Single Assessment Numeric Evaluation (SANE) score and Tegner Activity Level (TAL) Scale. Quality of life post-ACLR was evaluated using the Knee Injury and Osteoarthritis Outcome Score (KOOS). Implant safety was determined by analyzing ACLR failure rate and device and surgery-related adverse events.

Intervention and data collection

Participants underwent ACLR surgery utilizing one of the investigational devices, with the surgical intervention conducted by experienced orthopedic surgeons following standard protocols. The study utilized retrospective medical records for data collection, encompassing pre-operative assessments, intra-operative details, and post-operative follow-up conducted via telephonic call. Outcome measures, including functional recovery, patient-reported outcomes, activity levels, and quality-of-life assessments, were meticulously documented using standardized instruments during telephonic interviews. Adverse events and ACLR failure rates were assessed across the study duration.

Interpretation of scores

International Knee Documentation Committee

The IKDC questionnaire is a knee-specific, patient-reported outcome measure designed to assess overall knee function. This subjective scale evaluates the following three categories: symptoms, sports activities, and knee function. Scores are calculated by summing the individual item responses and then transforming the raw total into a scaled number ranging from 0 to 100. This final score represents knee function, with higher scores indicating better function. The IKDC subjective knee form score can be determined if responses are available for at least 90% of the items, which equates to at least 16 items [20].

Modified Cincinnati Rating System

The MCRS questionnaire comprises 12 questions, with eight contributing to the overall summary score. These scored questions address the areas of pain, swelling, function, and activity level. The total score is derived by summing the responses to these questions, where higher scores indicate excellent knee function and lower scores reflect poor knee function [20].

Tegner Activity Level Scale

The TAL Scale is designed to standardize the grading of work and sports activities. It features a graduated list of activities, ranging from daily living tasks to recreational and competitive sports. Patients choose the level that best reflects both their current activity and their activity before injury, using a scale from 0 to 10. A score of 0 indicates disability due to knee problems, while a score of 10 signifies participation in high-level activities [20].

Single Assessment Numeric Evaluation

SANE is a straightforward approach for assessing patients’ perceived functional improvement following meniscal repair surgery. It involves rating their current condition compared to their pre-injury baseline on a scale from 0 to 100. SANE scores are primarily utilized by orthopedic sports specialist surgeons, particularly for evaluating shoulder and knee conditions [21].

Knee Injury and Osteoarthritis Outcome Score

The KOOS is a self-administered tool that is valid, reliable, and responsive, making it suitable for both short-term and long-term follow-up of various knee injuries, including osteoarthritis. For this study, a subscale focusing on knee-related quality of life is used to evaluate patients’ quality of life after ACLR surgery. Higher scores indicate a better quality of life, while lower scores suggest a poorer quality of life [20].

Statistical analysis

Statistical analyses were conducted to derive meaningful insights from the collected data. Descriptive statistics were utilized to summarize the demographic data. The data were reported as percentages for qualitative variables and as the mean and standard deviation for quantitative variables. The data were confirmed to be normally distributed. SANE score and TAL scale were analyzed using the two-sample t-test.

Ethical consideration and quality control

Ethical considerations were of paramount importance throughout all stages of the study. Ethical approval was diligently obtained from the Ethics Committee, ensuring strict adherence to all relevant guidelines, including the Declaration of Helsinki and ISO 14155-2020. Before their inclusion in the study, all participants provided informed consent, thereby upholding the principles of autonomy and voluntary participation. To guarantee the highest standards of data integrity and quality control, a Contract Research Organization meticulously conducted regular quality control and assurance assessments, overseeing adherence to standardized operating procedures and ensuring the confidentiality and privacy of participants’ sensitive information in full compliance with regulatory requirements.

## Results

Table 1 provides an overview of subject disposition within the enrolled set, highlighting screening outcomes and enrollment status. A total of 49 subjects were screened for the study. Of these, two subjects experienced screen failure, resulting in a final enrollment of 47 subjects. Notably, all 47 enrolled subjects completed the study, reflecting a 100% study completion rate. It showcases effective subject recruitment, with a 96% enrollment rate, and strong study completion, evidenced by a 100% rate. Table 2 outlines the demographic characteristics of the enrolled set. The average age was 32.66 years, with a slight female predominance (61.7%). All participants were of Asian Indian ethnicity. These demographics provide crucial context for understanding the study population in the context of ACLR procedures.

**Table 1:** Subject’s disposition: enrolled set. N: number of subjects; %: percentage

Category	Overall, N (%)
Number of subjects screened	49
Number of subjects screen failure	2
Number of subjects enrolled	47
Number of subjects who completed the study	47 (100%)

**Table 2:** Summary of demographics: enrolled set. SD: standard deviation

Characteristics	Overall (N = 47)
Age (years)	
Mean	32.66
SD	9.88
Gender	
Female	29 (61.7%)
Male	18 (38.3%)
Race	
Asian Indian	47 (100.0%)

Table 3 highlights the usage of femoral and tibial fixation in 47 cases. The Proloop Adjustable Loop UHMWPE Suture Titanium Button 60 mm was the most used (57.4%), followed by the Infiloop Fixed Loop UHMWPE Suture Titanium Button 20 mm (40.4%) and the Infiloop Fixed Loop UHMWPE Suture Titanium Button 30 mm (2.1%). For tibial fixation, the T-Button A Adjustable Loop UHMWPE Suture PEEK Button 90 mm was exclusively used in all cases (100.0%). This indicates a preference for adjustable loop button 60 mm implants, emphasizing the importance of offering diverse options to meet surgical needs.

**Table 3:** Summary of implants used for femoral and tibial fixation. UHMWPE: ultra-high-molecular-weight polyethylene; N: number of subjects; PEEK: polyether ether ketone

Name of the implant	Overall (N = 47)
Femoral fixation	
Infiloop Fixed Loop UHMWPE Suture Titanium Button 20 mm	19 (40.4%)
Infiloop Fixed Loop UHMWPE Suture Titanium Button 30 mm	1 (2.1%)
Proloop Adjustable Loop UHMWPE Suture Titanium Button 60 mm	27 (57.4%)
Tibial fixation	
T-Button A Adjustable Loop UHMWPE Suture PEEK Button 90 mm	47 (100.0%)

Table 4 serves as a comprehensive repository of pivotal metrics pertaining to knee function and patient-reported outcomes post-ACLR. The data reveals a nuanced understanding of the efficacy of the surgical intervention, elucidating diverse facets of postoperative recovery. Notably, the IKDC score exhibited a mean value of 79.49 with a standard deviation (SD) of 12.67, indicative of the functional capacity and stability of the knee joint. Similarly, the MCRS score showed a mean of 81.32 with an SD of 11.57, underscoring the subjective appraisal of knee function post-surgery. Moreover, the SANE score for the affected joint/region demonstrated a mean of 77.83 with an SD of 11.11, while the SANE score for the opposite side exhibited a markedly higher mean of 93.83 with an SD of 9.15. Further, the TAL Scale, both before the injury and currently, showed mean values of 5.11 and 3.87, respectively, shedding light on the pre-injury activity level and the subsequent post-surgical modification. Lastly, the KOOS subscale score showed a mean of 83.81 with an SD of 13.07, delineating the broader impact of ACLR on various domains of knee health and quality of life. Collectively, these metrics offer a nuanced perspective on the functional outcomes and quality of life following ACLR surgery, while also highlighting areas of potential improvement and patient-specific variability.

**Table 4:** Postoperative summary of IKDC, MCRS, SANE, TAL score, and quality of life subscale from KOOS. The p-value is based on a two-sample t-test. *: significant p-value. IKDC: International Knee Documentation Committee (total range: 0 to 100); KOOS: Knee Injury and Osteoarthritis Outcome Score; MCRS: Modified Cincinnati Rating System Questionnaire (total range: 0 to 100); TAL: Tegner Activity Level scale (total range: 0 to 10); SANE: Single Assessment Numeric Evaluation (total range: 0 to 100); SD: standard deviation

Metric (n = 47)	Mean	SD	P-value
IKDC score	79.49	12.67	
MCRS score	81.32	11.57	
SANE score (affected joint/region)	77.83	11.11	<0.001*
SANE score (opposite side)	93.83	9.15	
TAL Scale (before injury)	5.11	1.07	<0.001*
TAL Scale (current)	3.87	0.99	
KOOS score	83.81	13.07	

Table 5 provides a comprehensive overview of the association between mean outcome scores, alongside the duration of follow-up. These findings underscore a trend of improvement over time, with higher IKDC and MCRS scores observed at greater than six months of follow-up (IKDC = 83.84 ± 9.15; MCRS = 84.76 ± 9.16), alongside improved SANE scores for both affected and opposite joints (affected joint/region = 73.61 ± 11.22; opposite side = 87.94 ± 10.88). Although minimal, a slight decrease in TAL Scale scores was noted from before injury to the current state (before injury = 4.89 ± 1.37; current: 3.72 ± 1.23), while KOOS scores indicated enhanced knee-related quality of life at greater than six months of follow-up (88.13 ± 10.19). With a mean total follow-up duration of 8.55 ± 4.79 months, these findings offer valuable insights into intervention efficacy and knee injury progression. There was no incidence of ACLR failure and device and surgery-related adverse events.

**Table 5:** Mean IKDC, MCRS, SANE, TAL, KOOS, and follow-up duration. Mean (SD) of total follow-up duration(in months): 8.55 ± 4.79 months. IKDC: International Knee Documentation Committee; KOOS: Knee Injury and Osteoarthritis Outcome Score; MCRS: Modified Cincinnati Rating System Questionnaire; TAL: Tegner Activity Level scale; SANE: Single Assessment Numeric Evaluation; SD: standard deviation

Duration of follow-up	IKDC mean score	MCRS mean score	SANE mean score (affected joint)	SANE mean score (opposite side)	TAL Scale mean score (before injury)	TAL Scale mean score (current)	KOOS mean score
Less than 6 months (n = 18)	72.48 ± 14.55	75.78 ± 13.09	73.61 ± 11.22	87.94 ± 10.88	4.89 ± 1.37	3.72 ± 1.23	76.83 ± 14.42
Greater than 6 months (n = 29)	83.84 ± 9.15	84.76 ± 9.16	80.45 ± 10.39	97.48 ± 5.43	5.24 ± 0.83	3.97 ± 0.82	88.13 ± 10.19

## Discussion

In the present investigation, a cohort of 47 patients underwent ACLR, with an average follow-up duration of 8.55 months. The mean age of the participants was 32.66 years, with a predominant representation of female individuals (61.7%). This demographic distribution aligns closely with findings reported in a previous study, where the mean age was 31.6 years, albeit with a male predominance of 83.9% [22]. The surgical approach in our study involved the utilization of various fixation methods, primarily the Proloop Adjustable Loop UHMWPE Suture Titanium Button 60 mm (57.4%), Infiloop Fixed Loop UHMWPE Suture Titanium Button 20 mm (40.4%), and Infiloop Fixed Loop UHMWPE Suture Titanium Button 30 mm (2.1%) for femoral fixation, while T-Button A Adjustable Loop UHMWPE Suture PEEK Button 90 mm was exclusively used for tibial fixation.

The ACLR procedure, acknowledged as the gold standard for restoring knee stability, significantly contributes to improved performance in daily activities [4]. Postoperative assessments, including functional outcomes, patient-reported outcomes, activity levels, and quality of life, revealed satisfactory results in the current study. Our primary aim was to evaluate postoperative functional outcomes in ACLR procedures utilizing various loop button implants. The mean IKDC score of 79.49 (±12.67) demonstrated diminished symptoms and enhanced sports activity and functional capacity. These findings were consistent with a previous study, indicating an improved postoperative mean IKDC score of 91.8 (±2.59) [22].

The demographic distribution aligns closely with findings reported in previous studies. Similar results were observed in a prospective case series where ACLR with PEEK screw showed improved IKDC score with a 24-month follow-up [23]. The comparison of single-bundle versus double-bundle reconstruction utilizing adjustable loop and interference screw fixation also showed significant improvement in the IKDC score of 75.3 ± 17.4 and 80.5 ± 13.6, respectively [24]. In comparison to our study, where the follow-up duration was shorter, the retrospective, observational study with a follow-up duration of 21.2 ± 14.2 months showed improvements in the IKDC scores [25].

The assessment of postoperative functional outcomes using the MCRS questionnaire revealed a commendable mean score of 81.32 (±11.57), signifying the exceptional capacity of patients to navigate daily activities. Parallels can be drawn to the findings of the earlier studies, which similarly reported favorable to outstanding outcomes in MCRS within a study spanning a 12-month follow-up period [26]. However, it is noteworthy that their study focused solely on patients with quadriceps and patellar tendon lesions, without exploring the variances in loop button types as we did in our investigation.

The assessment of patient-reported outcomes was conducted using the SANE scale, elucidating a mean score of 77.83 (±11.11) denoting the functional status of the affected joint or region of interest, showcasing a moderate level of function compared to the contralateral side (93.83 ± 9.15). The moderate to robust correlations detected between SANE and IKDC scores underscore the statistically significant elevation in SANE scores, thus reaffirming its efficacy as a tool for evaluating patient-reported outcomes. Notably, akin to our investigation, comparative analysis of patient-reported outcomes across different loop buttons was not undertaken in prior studies [27]. A registry-based study published in 2021 corroborated our findings, reporting a mean postoperative knee grade of 85.4 ± 14.2 (range = 20 to 100), although SANE score-based assessments were not conducted in that study, marking a deviation from our approach [28].

The assessment of quality of life via a subscale of the KOOS score revealed a mean KOOS score of 83.81 (±13.07). Consistent with our study, a comparative analysis of symptomatic and asymptomatic patients with primary unilateral ACLR showcased postoperative KOOS scores for quality of life of 55.0 (±15.4) and 81.8 (±11.8), respectively [29].

A study shared remarkable similarities with our study in terms of objectives. The study reported postoperative IKDC (80.6 ± 16.7) and KOOS quality of life (78.8 ±23.9) scores, which closely mirrored our findings [30]. However, the sample size in our study may limit its generalizability to the broader population.

Our study reported a 0% failure rate post-ACLR, with no device- or surgery-related adverse events noted. This insight is bolstered by recent evidence published in 2023, highlighting a comparison between over-the-top and transportal drilling techniques in patients undergoing surgery for failed revision ACLR, where notably high failure and complications were observed [31].

The extrapolation of our study findings to a broader population is hindered by several limitations. These include a limited sample size, a retrospective study design, and a shortened follow-up period. In contrast to studies with longer observation durations, our study had a comparatively shorter follow-up duration. Furthermore, the incorporation of diverse reconstruction techniques poses challenges in discerning outcomes attributable to specific interventions.

## Conclusions

The study has delivered significant insights into ACLR procedures through a comprehensive comparison of various loop buttons for femoral and tibial fixation. Positive outcomes across multiple assessments, including IKDC, MCRS, TAL Scale, and the quality of life subscale of KOOS, underscore the effectiveness of interventions such as the T-Button-A Adjustable Loop UHMWPE Suture PEEK Button, Infiloop Fixed Loop UHMWPE Suture Titanium Button, and Proloop Adjustable Loop UHMWPE Suture Titanium Button in ACLR. The absence of ACLR failures and adverse effects from devices or surgeries further reinforces the promise of these interventions. Nevertheless, advocating for randomized clinical trials with larger cohorts remains essential to validate these findings conclusively.
